# Efficacy of Transcutaneous Auricular Trigeminal Nerve Stimulation in Migraine: A Randomized, Participant- and Assessor-Blinded, Sham-Controlled Trial

**DOI:** 10.3390/jcm15218395

**Published:** 2026-10-28

**Authors:** Semiha Yenişehir, Alper Perçin, Ali Veysel Özden

**Affiliations:** 1 Department of Physiotherapy and Rehabilitation, Faculty of Health Sciences, Ordu University, 52200 Ordu, Türkiye; 2 Department of Therapy and Rehabilitation, Şiran Dursun Keleş School of Health Services, Gümüşhane University, 29100 Gümüşhane, Türkiye; alperpercin@yahoo.com; 3 Department of Physiotherapy and Rehabilitation, Health Services Vocational School, Bahcesehir University, 34353 Istanbul, Türkiye; aliveysel.ozden@bau.edu.tr

**Keywords:** migraine disorders, pain measurement, transcutaneous electric nerve stimulation, trigeminal nerve, randomized controlled trial

## Abstract

**Background:** Migraine is a leading cause of disability worldwide. Transcutaneous auricular trigeminal nerve stimulation (taTNS) has emerged as a non-invasive neuromodulation approach targeting trigeminal pathways involved in pain modulation. **Objective**: To investigate the effects of taTNS on migraine pain intensity, sleep quality, disability, and Heart Rate Variability (HRV) in patients with migraine. **Materials and Methods:** This randomized, participant- and assessor-blind, sham-controlled trial included 102 participants who received taTNS (*n* = 51) or sham taTNS (*n* = 51). Active taTNS was administered bilaterally at 60 Hz, 250 μs, and up to 16 mA for 20 min, three times weekly for 12 weeks; sham stimulation used identical electrode placement at 0 mA. The primary outcome measures were migraine pain intensity (VAS), migraine-related disability (MIDAS) and sleep quality (PSQI); secondary outcomes included HRV, assessed using the standard deviation of normal-to-normal intervals (SDNN) and root mean square of successive differences (RMSSD). **Results:** At post-intervention, VAS and MIDAS scores were significantly lower in the taTNS group than in the sham-taTNS group (both *p* < 0.001), whereas PSQI scores did not differ significantly between groups (*p* = 0.870). RMSSD was significantly higher in the taTNS group than in the sham-taTNS group (*p* = 0.013), whereas the between-group difference in SDNN was not statistically significant (*p* = 0.187). **Conclusions:** Compared with sham stimulation, taTNS was associated with greater reductions in migraine pain intensity and migraine-related disability, but no significant between-group difference was observed in sleep quality.

## 1. Introduction

Migraine is a recurrent neurological disorder characterized by moderate to severe, often unilateral and pulsating headache attacks, typically lasting 4–72 h and frequently accompanied by nausea, photophobia, and phonophobia [1]. According to the Global Burden of Disease Study 2023, migraine accounted for 40.9 million years lived with disability (YLDs) worldwide and ranked eighth among all conditions in terms of age-standardized YLD rates (487.5 per 100,000 population) [2]. Migraine adversely affects social and family life; it reduces quality of life by causing incapacity and a decline in sleep quality, and also imposes an economic burden through absenteeism from work [3,4,5].

The pathophysiology of migraine is associated with the activation and sensitisation of the trigemino-vascular system and the central modulation of cranial pain pathways [6]. Glutamate, which plays a central role in the pathogenesis of migraine, activates the ascending trigeminal and trigemino–thalamic pathways, whilst the calcitonin gene-related peptide released from trigeminal afferents triggers neurogenic inflammation and pain transmission [7]. Repeated activation of these pathways may lead to cortical hyperexcitability, peripheral and central sensitisation, and the progression of migraine to a chronic state [8]. Trigeminal nerve stimulation may modulate brainstem and forebrain structures involved in pain processing. The auriculotemporal nerve, originating from the mandibular division (V3) of the trigeminal nerve, supplies sensory innervation to portions of the external ear, including regions near the base of the helix, and may contribute to vasomotor and parasympathetic regulation [9,10].

Migraine is frequently associated with autonomic dysfunction, including alterations in sympathetic and parasympathetic nervous system activity [11]. Heart rate variability (HRV) is a well-established, non-invasive measure of autonomic nervous system (ANS) activity and has been proposed as a biomarker of pain-related disorders [11]. Chuang et al. found that, compared with a healthy control group, migraine patients had lower values for standard deviation of normal-to-normal intervals (SDNN), root mean square of successive differences (RMSSD), high-frequency power (HF), and total power, suggesting altered autonomic regulation in migraine [11].

The laterality of auricular stimulation is another factor that must be considered in neuromodulation protocols. The external ear receives overlapping sensory innervation from various nerves, including the auriculotemporal branch of the trigeminal nerve, the auricular branch of the vagus nerve, and the cervical nerves. Furthermore, the trigeminal and vagal afferent pathways exhibit bilateral central projections and integration; this provides a theoretical neurophysiological basis for stimulation on both sides or on one side [12]. In this context, bilateral auricular stimulation may allow for the integration of sensory input from both ears. However, direct evidence demonstrating the superiority of bilateral auricular stimulation over unilateral stimulation in terms of migraine-related pain modulation remains insufficient, and the optimal stimulation laterality has not yet been determined. Stimulation frequency has been investigated in various neuromodulation approaches for migraine treatment. Straube et al. (2015) compared 1 Hz and 25 Hz auricular vagus nerve stimulation and reported a greater reduction in headache days with 1 Hz in the protocol-adherence analysis [13]; however, this difference was not found to be statistically significant in the intention-to-treat analysis. In contrast, external trigeminal nerve stimulation at 60 Hz has been used for migraine treatment; the proposed mechanism involves the modulation of trigemino-vascular nociceptive transmission via the spinal trigeminal nucleus [14]. Furthermore, randomized, placebo-controlled trials using 100 Hz external trigeminal stimulation have demonstrated improvements in acute migraine pain [15,16]. However, these studies involve different anatomical targets and stimulation protocols and do not establish the superiority of high- or low-frequency stimulation in terms of migraine-related pain modulation.

While transcutaneous auricular vagus nerve stimulation (taVNS) has been studied in migraine [13,17], to our knowledge, the effects of transcutaneous auricular trigeminal nerve stimulation (taTNS) on headache severity, sleep quality, disability, and HRV in individuals with migraine have not been comprehensively investigated. Therefore, this study aimed to investigate the effects of taTNS on migraine pain intensity, sleep quality, disability level, and HRV in patients with migraine.

## 2. Materials and Methods

### 2.1. Study Design and Population

This randomized, parallel-group, participant- and assessor-blinded, sham-controlled trial was conducted in patients diagnosed with migraine at the Physiotherapy and Rehabilitation Clinic of Avrasya University between January and June 2026. The trial was conducted in accordance with the Declaration of Helsinki, and its reporting followed the Consolidated Standards of Reporting Trials (CONSORT) guidelines (Table S1). All participants provided informed consent before taking part in the study. Ethical approval was granted by the Clinical Research Ethical Board of Karadeniz Teknik University (date: 13 August 2025, protocol number: 2025/5). The trial was registered at ClinicalTrials.gov under the identifier NCT07457268.

### 2.2. Sample Size and Participants

The sample size was calculated a priori using G*Power software (version 3.1.9.6) for a repeated-measures ANOVA with a within–between interaction. Assuming an effect size of f = 0.20, an alpha level of 0.05, a statistical power of 95%, two groups, and two measurement time points, the minimum required sample size was calculated as 84 participants. Allowing for an anticipated dropout rate of 15%, the target sample size was increased to 100 participants (50 per group).

The inclusion criteria were (1) aged 18–45 years; (2) literate in Turkish; (3) diagnosed with episodic migraine without aura according to the International Classification of Headache Disorders (third edition); and (4) experiencing at least two migraine days per month during the baseline period. The exclusion criteria were (1) being pregnant or breastfeeding; (2) regular use of analgesics or non-steroidal anti-inflammatory drugs for more than 15 days per month; (3) pacemaker implantation; (4) a history of addiction, syncope, or abnormal ECG findings; (5) a history of intracranial hemorrhage or significant head trauma; (6) a diagnosis of epilepsy, severe anxiety, or depression; (7) other types of headaches, including paresthesia or medication-overuse headaches; and (8) cognitive, visual, or auditory problems that would prevent participation in the study.

### 2.3. Randomization and Blinding

**Figure 1:**
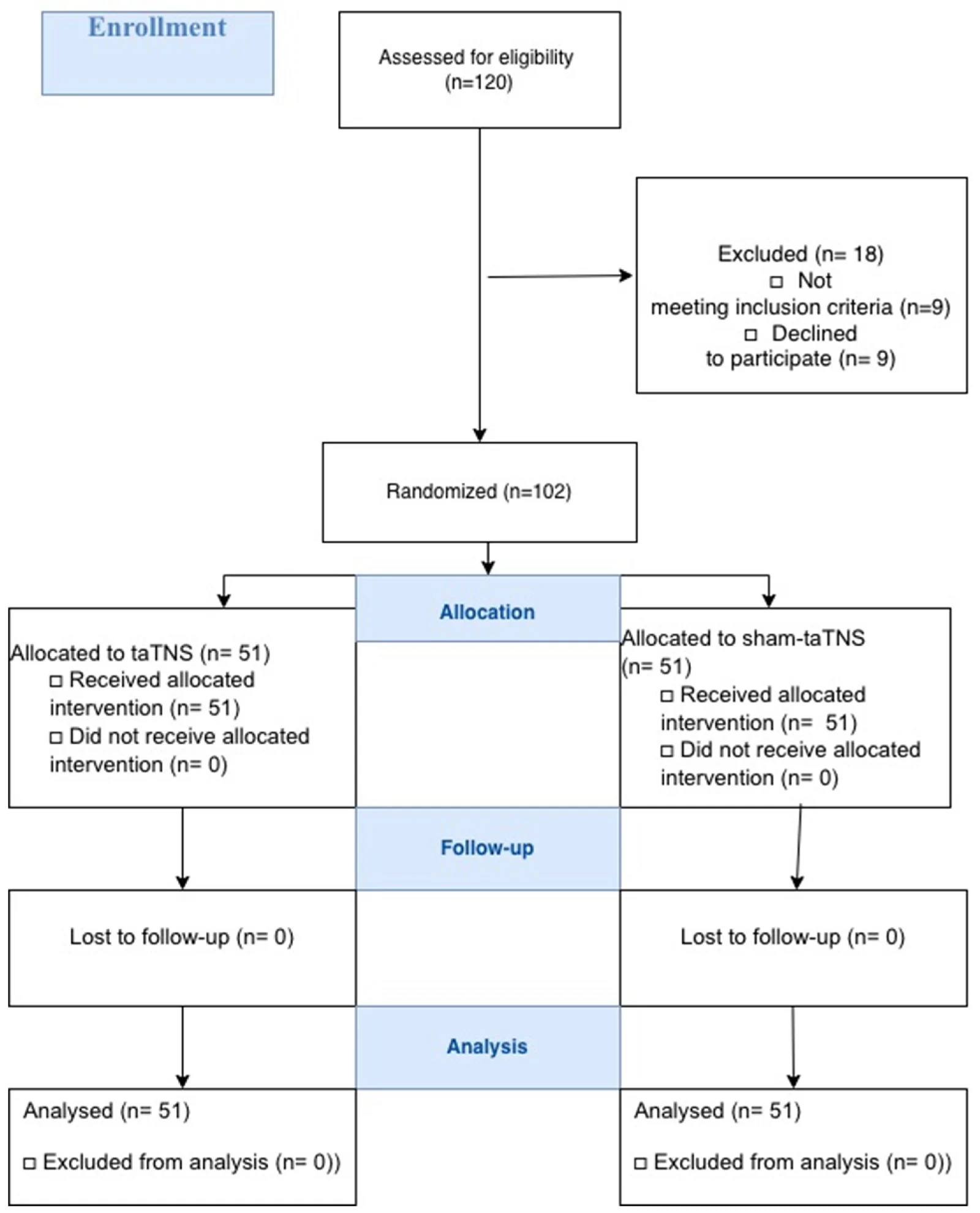
Flow diagram of the participants.

Participants were randomly allocated in a 1:1 ratio to either the taTNS group or the sham-taTNS (control) group using a computer-generated randomization sequence (https://www.randomizer.org/). Investigators responsible for participant recruitment and enrollment did not have access to the allocation sequence. Participants assigned to the intervention group received taTNS, whereas those assigned to the control group received sham-taTNS (Figure 1).

The physiotherapist administering the stimulation was not blinded, whereas the participants and the outcome assessor were blinded to group allocation. Participants were informed that they had an equal chance of being assigned to either group but were not provided with information regarding their allocation. To maintain participant blinding, interventions were administered with identical electrodes individually in separate rooms to prevent communication among participants about their treatment experiences. The session durations were also planned in such a way as to minimize information sharing between the taTNS and sham-taTNS groups. The physiotherapist who carried out the intervention did not explain the group allocation to the participants.

To assess the success of the blinding method, at the end of the treatment period, participants were asked to guess which group (intervention or control) they had been assigned to. They were also asked to rate their confidence in these guesses using a 0–10 cm Visual Analog Scale (VAS), where 0 indicated no confidence at all and 10 indicated complete confidence.

### 2.4. Interventions

**Figure 2:**
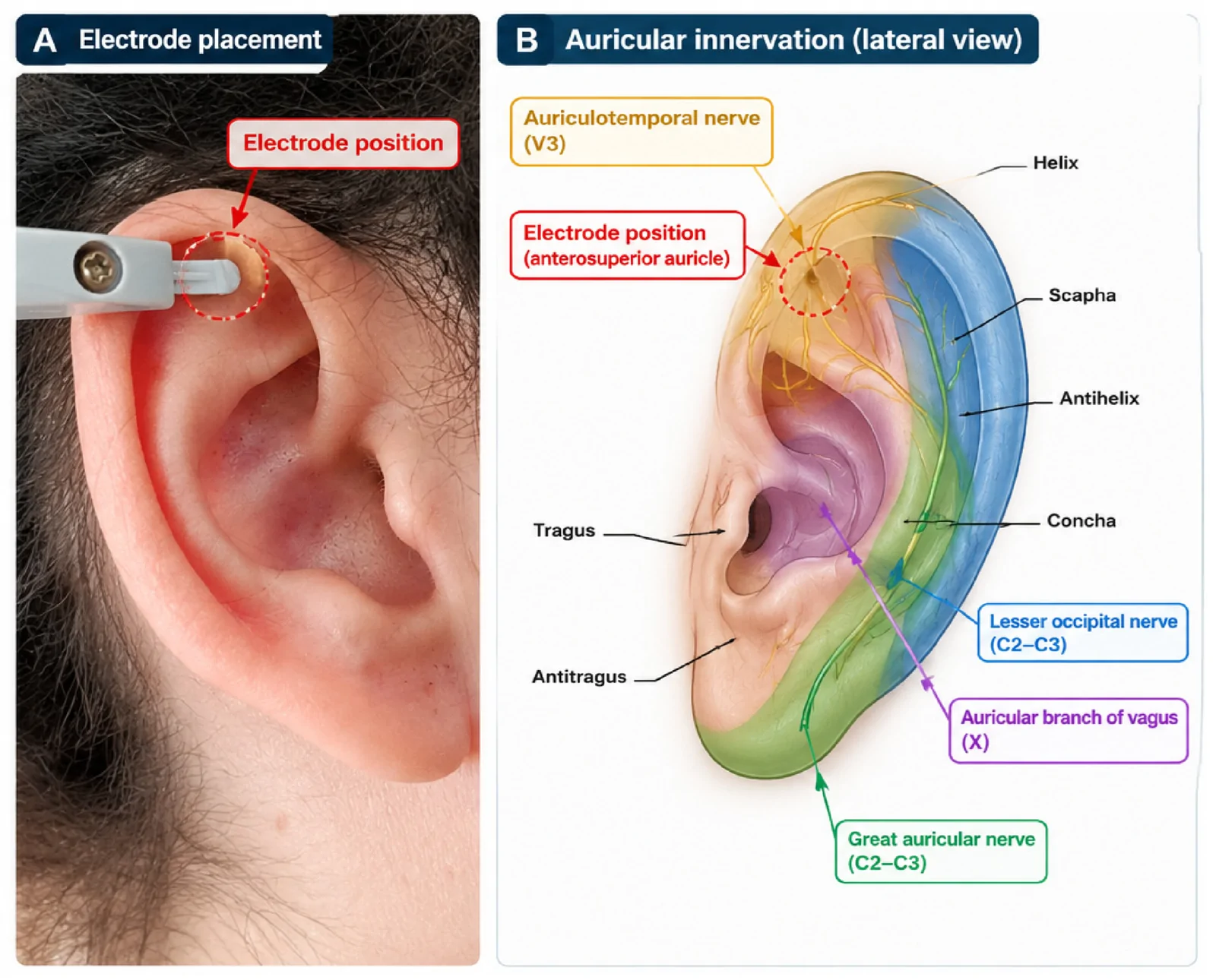
(**A**) Electrode placement in the study. (**B**) Sensory innervation of the auricle and the electrode location.

taTNS and sham-taTNS interventions were administered by a physiotherapist. Participants were positioned in long sitting with back support. Stimulation was delivered using a dual-channel Vagustim device (Vagustim Health Technologies, San Francisco, CA, USA, Registration Number: 9616877). In both groups, clip-on bipolar electrodes were placed on the upper-anterior part of the auricle (Figure 2). This site was selected to target the auriculotemporal nerve (ATN), a sensory branch of the mandibular division (V3) of the trigeminal nerve, based on its reported distribution in the anterior–superior auricular region [10]. However, as the distribution of sensory nerves in the auricle is heterogeneous and overlapping, the possibility of simultaneous stimulation of nerve fibers other than the adjacent trigeminal nerve must not be overlooked [18]. Electrode gel was used to facilitate electrical conduction. The stimulation parameters were based on a previously reported transcutaneous trigeminal nerve stimulation protocol [19]. Bilateral auricular stimulation was delivered using a biphasic rectangular current with a pulse width of 250 μs, a frequency of 60 Hz, and a stimulation duration of 20 min, with the current intensity increased to a maximum of 16 mA [20]. The intervention was administered three times per week for 12 weeks.

Sham-taTNS was performed to mimic the active intervention without delivering electrical stimulation. The electrodes were placed on the same anatomical regions as in the active taTNS application. During the procedure, the current intensity was 0 mA and the device was switched on.

### 2.5. Outcome Measures

The physical (age, gender, height, body weight) characteristics of participants were recorded. All outcome measures were assessed at baseline and after completion of the 12-week intervention. The primary outcomes were pain intensity assessed using the VAS, migraine-related disability assessed using the Migraine Disability Assessment Scale (MIDAS), and sleep quality assessed using the Pittsburgh Sleep Quality Index (PSQI). HRV, assessed using the standard deviation of normal-to-normal intervals (SDNN) and the root mean square of successive differences (RMSSD), was evaluated as a secondary outcome.

The MIDAS is a validated instrument for evaluating headache-related disability in individuals with migraine [21,22]. The validity and reliability of its Turkish adaptation were established by Ertaş et al. [23]. The questionnaire comprises five items that assess the impact of migraine on daily functioning over the preceding three months. Participants report the number of days affected by headache across three domains: school or employment, household responsibilities, and family, social, or recreational activities. In addition to days when activities could not be performed, the instrument captures days on which productivity in paid work or household tasks was reduced by at least 50% [23]. Disability severity was classified according to the total MIDAS score as follows: 0–5, little or no disability; 6–10, mild disability; 11–20, moderate disability; and scores above 21, severe disability [23].

Buysse et al. [24] developed PSQI to assess quality of sleep [24]. The validity and reliability of the Turkish version of the PSQI have been demonstrated [25]. It consists of a total of 24 items, 19 of which are self-reported. Five questions are completed by a bed partner or roommate for clinical information only and are not included in scoring. Item 19 is self-reported but not included in the global score calculation. A total score >5 indicates poor sleep quality [24,26].

HRV was measured using the Polar H10 device (Copyright 2016 Polar Electro Oy, FI-90440 Kempele, Finland, Registration Number: 3003290656). The Polar H10 device was placed at heart level using a chest strap, the data obtained from this device were synchronized and transferred to the Polar Flow application (© Polar Electro, 2024) via a Bluetooth-connected iPhone SE (third generation). The data transferred to the Polar Flow app were analyzed using the Kubios HRV Standard (version 3.5.0) (www.kubios.com).

Short-term HRV recordings were obtained over five minutes, as this approach was considered more feasible than continuous 24 h monitoring [27]. The five-minute recording duration was also selected in accordance with recommendations concerning the reliability of short-term HRV assessment [28]. Measurements were conducted with participants seated in a quiet room maintained at 20–22 °C (68–77 °F).

Two time-domain HRV indices were examined: SDNN, representing overall variability in normal-to-normal cardiac intervals, and RMSSD, reflecting short-term fluctuations between consecutive normal heartbeats and predominantly associated with cardiac parasympathetic regulation [29,30].

### 2.6. Statistical Analysis

Statistical analyses were performed using the Statistical Package for the Social Sciences (SPSS, version 25.0 for Mac). Descriptive data are presented as mean ± standard deviation (SD) or number and percentage. The distribution of continuous variables was evaluated using the Shapiro–Wilk test and Q–Q plots.

For each outcome variable, a repeated measures analysis of variance (ANOVA) was conducted, with time (baseline and post-intervention) as the within-subjects factor and group (taTNS and sham-taTNS) as the between-subjects factor. Time × group interactions and partial eta-squared (ηp^2^) effect sizes were analyzed. For the primary outcomes (VAS, MIDAS and PSQI), the Holm procedure was applied to the *p*-values of the time × group interactions to correct for multiple comparisons.

Independent-sample *t*-tests were additionally used to describe between-group differences at baseline and post-intervention, whereas paired-samples *t*-tests were used to describe within-group changes from baseline to post-intervention. These comparisons were considered complementary to the principal time × group interaction analyses.

The minimal clinically important difference (MCID) was estimated using the distribution-based 0.5 × baseline SD method. Cohen’s d was calculated to quantify the magnitude of within-group changes and was interpreted as small (0.2), medium (0.5), large (0.8), and very large (≥1.2). All statistical significance was set at *p* < 0.05.

## 3. Results

### 3.1. Participants

As shown in Figure 1, 120 patients with migraine were assessed for eligibility, of whom 18 were excluded. A total of 102 participants (65 women and 37 men) were randomized to the taTNS (*n* = 51) and sham-taTNS (*n* = 51) groups. All randomized participants completed the 12-week intervention and post-intervention assessment, with no loss to follow-up. Missed sessions were rescheduled to ensure completion of all 36 prescribed sessions.

The mean age of the total sample was 35.95 ± 7.06 years, and the mean BMI was 24.57 ± 2.30 kg/m^2^. Baseline physical characteristics did not differ significantly between the groups (Table 1).

### 3.2. Pain Intensity, Migraine-Related Disability, and Sleep Quality

At baseline, VAS, MIDAS, and PSQI scores did not differ significantly between the groups. Significant time × group interactions were observed for VAS (F = 51.925, *p* < 0.001, ηp^2^ = 0.342) and MIDAS (F = 56.169, *p* < 0.001, ηp^2^ = 0.360), whereas the interaction for PSQI was not significant (F = 1.478, *p* = 0.227, ηp^2^ = 0.015). At post-intervention, VAS and MIDAS scores were significantly lower in the taTNS group than in the sham-taTNS group (*p* < 0.001), whereas PSQI did not differ significantly between the groups (*p* = 0.870). The between-group difference in change from baseline to post-intervention was −1.92 cm (95% CI: −2.45 to −1.39) for VAS and −3.45 points (95% CI: −4.36 to −2.54) for MIDAS, indicating greater reductions in pain intensity and migraine-related disability in the taTNS group compared with the sham-taTNS group. After Holm correction for multiple testing, the time × group interactions remained significant for VAS and MIDAS but not for PSQI (Table 2).

### 3.3. Heart Rate Variability

Significant time × group interactions were observed for SDNN (F = 18.951, *p* < 0.001, ηp^2^ = 0.159) and RMSSD (F = 10.217, *p* = 0.002, ηp^2^ = 0.093). At post-intervention, RMSSD was significantly higher in the taTNS group than in the sham-taTNS group (*p* = 0.013), whereas the between-group difference in SDNN was not significant (*p* = 0.187) (Table 2).

The reduction in VAS scores in the taTNS group (Δ = 2.11) exceeded the estimated MCID threshold of 0.88. Similarly, the reduction in MIDAS scores (Δ = 3.49) exceeded the estimated MCID threshold of 1.54. In contrast, changes in PSQI (Δ = 0.04), SDNN (Δ = 0.98), and RMSSD (Δ = 0.39) did not exceed their respective estimated MCID thresholds (1.90, 2.00, and 1.15). Thus, despite statistically significant time × group interactions for SDNN and RMSSD, the magnitude of the within-group changes in these HRV parameters did not exceed the estimated MCID thresholds.

### 3.4. Adverse Effects

The taTNS intervention was well tolerated, with no side effects reported by participants.

### 3.5. Assessment of Participant Blinding

At the end of the intervention period, 37 of 51 participants (72.55%) in the taTNS group correctly identified their allocation, compared with 16 of 51 participants (31.37%) in the sham-taTNSgroup. Exact binomial tests indicated that the proportion of correct guesses differed significantly from the chance level of 50% in both the taTNS group (*p* = 0.002) and the sham-taTNS group (*p* = 0.011). Bang’s Blinding Index was 0.45 for the taTNS group and −0.37 for the sham-taTNS group, indicating a tendency toward correct identification in the active group and toward incorrect identification in the sham-taTNS group. These findings suggest that participant blinding was not fully maintained, particularly in the active stimulation group.

## 4. Discussion

This study has shown that taTNS was associated with greater reductions in migraine pain intensity and migraine-related disability compared with sham stimulation. No changes were observed in sleep quality. Although changes in HRV parameters indicate a measurable autonomic response to the intervention, these changes did not exceed the predicted threshold values for clinical significance. These findings suggest that the clinical effects of the intervention are more pronounced in terms of pain intensity and migraine-related disability than in terms of autonomic outcomes.

Trigeminal afferent activation contributes to migraine pain via the release of vasoactive neuropeptides, neurogenic inflammation, and peripheral and central sensitisation [7,31]. For this reason, the modulation of trigeminal afferent signal transmission represents one of the possible mechanisms by which peripheral electrical stimulation may influence migraine-related pain. Previous studies on trigeminal neuromodulation have also demonstrated beneficial effects on pain; however, fundamental differences in the patient population and stimulation approaches limit direct comparison with this study [32].

Consequently, the reduction in migraine pain intensity and migraine-related disability observed in this study may be consistent with the modulation of trigeminal nociceptive pathways; however, it does not conclusively prove a mechanism specific to the trigeminal nerve. The nucleus tractus solitarius (NTS) represents another potential pathway, as vagal afferent fibers project primarily to the NTS, which is connected to several brainstem regions involved in nociceptive processing and autonomic regulation, including the spinal trigeminal nucleus and locus coeruleus [33]. Given the complex and overlapping sensory innervation of the external ear, concurrent involvement of trigeminal and vagal pathways cannot be excluded. Therefore, the current findings should not be interpreted as evidence of selective trigeminal nerve activation.

The sensory innervation of the external ear is complex and involves the auriculotemporal branch of the trigeminal nerve, the auricular branch of the vagus nerve and the cervical nerves [10,33]. Anatomical studies have shown that the distribution areas of the auricular nerve are heterogeneous and variable; this limits the ability to identify selective nerve involvement based solely on electrode placement [10,33]. Consequently, although the intervention in the present study was intended to target an auricular region with trigeminal innervation, concomitant recruitment of vagal or other auricular afferents cannot be excluded.

This is particularly important, as taVNS has previously been studied in relation to migraines [13,17]. Auricular stimulation can influence the brainstem and higher-level networks involved in nociceptive processing via different or converging afferent pathways [13,17]. For this reason, the clinical effects observed in this study may reflect a combination of simultaneous vagal activation and modulation of trigeminal afferent signals, or other auricular afferent input. To clarify the relative contributions of these pathways, there is a need for future studies that directly compare the areas of stimulation and include neurophysiological or neuroimaging measures of activity in the target region.

Poor sleep quality was found in both groups at baseline and following the intervention. No significant time × group interaction was observed for PSQI. Poor sleep quality is common among individuals with migraine and has been associated with migraine occurrence and burden [34]. Sleep is influenced by multiple psychological, behavioral, environmental, and physiological factors, which may limit the effect of a single neuromodulatory intervention on global subjective sleep quality. Although acute trigeminal neuromodulation has been investigated in relation to sleep and alertness, previous evidence has not established a consistent beneficial effect on sleep [35]. In addition, the use of a self-reported questionnaire in the present study may have limited the detection of subtle physiological changes in sleep. Future studies incorporating objective measures such as actigraphy or polysomnography may provide additional information.

Autonomic dysfunction and alterations in HRV have been reported in individuals with migraine [11,36]. In the present study, significant time × group interactions were observed for SDNN and RMSSD corresponding to large and moderate interaction effects. SDNN increased significantly within the active stimulation group, although the post-intervention between-group difference was not statistically significant. RMSSD increased significantly within the active stimulation group and was significantly higher than in the sham group at the post-intervention assessment. Previous work by Perçin et al. also demonstrated autonomic effects following taTNS in healthy individuals, including changes in RMSSD; however, differences in the study population and stimulation protocol limit direct comparison with the present findings [9].

RMSSD predominantly reflects short-term cardiac parasympathetic modulation and is frequently used as a time-domain measure of vagally mediated HRV [28,29,37]. Nevertheless, changes in HRV cannot identify which auricular nerve was responsible for the observed autonomic response. The HRV findings therefore should not be considered evidence of selective trigeminal engagement and are also compatible with concomitant vagal afferent recruitment [33]. Furthermore, although statistically significant interaction effects were observed for SDNN and RMSSD, the absolute changes in these values did not exceed the estimated MCID thresholds used in this study. Therefore, these findings should not be interpreted as evidence of a clinically meaningful improvement in autonomic function.

### Strengths and Limitations

The randomized, sham-controlled design, blinded outcome assessment, and simultaneous evaluation of clinical and physiological outcomes are strengths of the present study. However, several limitations should be considered when interpreting the findings.

The International Headache Society guidelines recommend migraine-day and headache-day outcomes for clinical trials of neuromodulation devices used for the preventive treatment of migraine [38]. Therefore, the present findings should be interpreted as evidence of changes in the measured outcomes of pain intensity and migraine-related disability rather than as a comprehensive assessment of preventive migraine efficacy. Migraine pain intensity was assessed using the VAS rather than repeated measurements throughout individual migraine attacks. Given the episodic and fluctuating nature of migraine, this approach may not fully capture within-day variations or peak attack intensity. Future studies should incorporate prospective headache diaries and repeated pain assessments to provide a more comprehensive evaluation of changes in migraine pain intensity. Second, participant blinding may not have been fully maintained in the taTNS group, as 72.55% of participants correctly identified their treatment allocation. In contrast, most participants in the sham-taTNS group believed that they had received active stimulation. Therefore, the possibility that treatment expectations influenced subjective outcomes, particularly in the active group, cannot be excluded. Third, selective activation of the auriculotemporal branch of the trigeminal nerve was not directly verified. Given the complexity and variability of sensory innervation of the external ear [10,33], concomitant stimulation of vagal or other auricular afferents cannot be excluded. Finally, the study did not include long-term follow-up, and sleep quality was assessed using a self-reported questionnaire rather than objective sleep measures. In addition, only individuals with episodic migraine without aura were included, which may limit the generalizability of the findings to other migraine phenotypes.

## 5. Conclusions

The taTNS intervention was associated with reductions in pain intensity and migraine-related disability compared with sham stimulation in individuals with episodic migraine without aura. However, these findings should be interpreted cautiously because incomplete participant blinding may have influenced self-reported outcomes. Although statistically significant changes were observed in HRV parameters, these changes did not exceed the estimated MCID thresholds, and their clinical relevance remains uncertain. No significant improvement in sleep quality was observed. Furthermore, selective trigeminal nerve engagement was not directly demonstrated, and the findings should not be interpreted as evidence of an exclusively trigeminal mechanism.

## Figures and Tables

**Table 1:** Baseline characteristics of the participants.

	taTNS *n* = 51	Sham-taTNS *n* = 51	*p*	t	95%CI Lower	95%CI Upper
	Mean ± SD	Mean ± SD				
Age (years)	36.63 ± 6.73	35.27 ± 7.39	0.336	0.967	−1.423	4.129
Height (cm)	167.27 ± 9.29	166.94 ± 9.68	0.860	0.177	−3.395	4.061
Weight (kg)	69.27 ± 13.16	68.49 ± 12.43	0.758	0.310	−4.243	5.812
BMI (kg/m^2^)	24.61 ± 3.16	24.53 ± 2.85	0.898	0.128	−1.1061	1.2591

BMI: Body mass index; taTNS: Transcutaneous auricular trigeminal nerve stimulation; CI: Confidence Interval; SD: Standard deviation.

**Table 2:** Within- and between-group comparisons of clinical and heart rate variability outcomes.

Outcome	taTNS (*n* = 51)			Sham-taTNS (*n* = 51)			Between-Group *p*		Time × Group Interaction
	Baseline	Post	Within-Group *p*	Baseline	Post	Within-Group *p*	Baseline	Post	
VAS (cm)	7.35 ± 1.76	5.24 ± 1.57	<0.001 *	7.39 ± 1.72	7.20 ± 1.61	0.067	0.910	<0.001 *	F = 51.925; *p* < 0.001 *; ηp^2^ = 0.342
MIDAS	15.22 ± 3.07	11.73 ± 3.58	<0.001 *	15.33 ± 3.08	15.29 ± 3.04	0.735	0.847	<0.001 *	F = 56.169; *p* < 0.001 *; ηp^2^ = 0.360
PSQI	14.33 ± 3.79	14.29 ± 3.45	0.727	14.51 ± 3.84	14.18 ± 3.78	0.127	0.816	0.870	F = 1.478; *p* = 0.227; ηp^2^ = 0.015
SDNN	19.15 ± 3.97	20.13 ± 4.16	<0.001 *	19.12 ± 3.26	19.15 ± 3.25	0.117	0.958	0.187	F = 18.951; *p* < 0.001 *; ηp^2^ = 0.159
RMSSD	10.41 ± 2.30	10.80 ± 2.49	0.003 *	9.41 ± 3.10	9.40 ± 3.07	0.569	0.068	0.013 *	F = 10.217; *p* = 0.002 *; ηp^2^ = 0.093

taTNS: transcutaneous auricular trigeminal nerve stimulation, VAS: visual analog scale, MIDAS: Migraine Disability Assessment Scale, PSQI: Pittsburgh Sleep Quality Index, SDNN: standard deviation of normal-to-normal intervals, RMSSD: root mean square of successive differences, SD: standard deviation, ηp^2^: partial eta squared. * *p* < 0.05.

## Data Availability

Data are available from the corresponding author upon reasonable request.

## Supplementary Materials

The following supporting information can be downloaded at: https://www.mdpi.com/article/10.3390/jcm15218395/s1, Table S1: The CONSORT reporting checklist.
